# Electrodeposition of crystalline silicon films from silicon dioxide for low-cost photovoltaic applications

**DOI:** 10.1038/s41467-019-13065-w

**Published:** 2019-12-18

**Authors:** Xingli Zou, Li Ji, Jianbang Ge, Donald R. Sadoway, Edward T. Yu, Allen J. Bard

**Affiliations:** 1 Center for Electrochemistry, Department of Chemistry, The University of Texas at Austin, Austin, TX 78712 USA; 2 State Key Laboratory of Advanced Special Steel & Shanghai Key Laboratory of Advanced Ferrometallurgy & School of Materials Science and Engineering, Shanghai University, Shanghai, 200444 China; 3 Microelectronics Research Center, Department of Electrical and Computer Engineering, The University of Texas at Austin, Austin, TX 78712 USA; 4 State Key Laboratory of ASIC and System, School of Microelectronics, Fudan University, Shanghai, 200433 China; 5 Department of Materials Science and Engineering, Massachusetts Institute of Technology, Cambridge, MA 02139 USA

**Keywords:** Electrical and electronic engineering, Materials for energy and catalysis, Solar cells

## Abstract

Crystalline-silicon solar cells have dominated the photovoltaics market for the past several decades. One of the long standing challenges is the large contribution of silicon wafer cost to the overall module cost. Here, we demonstrate a simple process for making high-purity solar-grade silicon films directly from silicon dioxide via a one-step electrodeposition process in molten salt for possible photovoltaic applications. High-purity silicon films can be deposited with tunable film thickness and doping type by varying the electrodeposition conditions. These electrodeposited silicon films show about 40 to 50% of photocurrent density of a commercial silicon wafer by photoelectrochemical measurements and the highest power conversion efficiency is 3.1% as a solar cell. Compared to the conventional manufacturing process for solar grade silicon wafer production, this approach greatly reduces the capital cost and energy consumption, providing a promising strategy for low-cost silicon solar cells production.

## Abstract (web summary)

The photovoltaics market has been dominated by crystalline silicon solar cells despite the high cost of the silicon wafers. Here Zou et al. develop a one-step electrodeposition process in molten salt to produce high-purity solar-grade silicon films, delivering power conversion efficiency of 3.1%.

## Introduction

Currently, global energy generation still depends strongly on fossil fuels^1^. Driven by the rapidly increasing energy demands and the negative environmental impact of fossil fuels, renewable energy has attracted tremendous attention in recent decades. Solar cells, utilizing sunlight to generate electricity directly, have been recognized as one of the most promising technologies for solving the energy issues^1–12^. Crystalline-silicon solar cells have dominated the photovoltaics market for the past several decades and are most likely to continue to be the primary technology for the photovoltaics industry in the future due to its abundant raw materials supply and non-toxicity^1,6^. To make silicon-based solar cells more competitive, improving the power conversion efficiency and decreasing the module costs are the most direct routes. For efficiency enhancement, innovative cell architectures involving complex processing procedures are usually required, often resulting in increased overall cost^13^. Reducing the silicon production cost and silicon material usage thus offers an alternative route for continued growth of crystalline-silicon photovoltaics technology in the future.

One of the largest contributions to overall module manufacturing cost still comes from silicon wafer production, involving complex processing and intensive energy consumption due to the high temperature requirement for silicon crystallization process^13^. To address this problem, direct production of silicon at low temperature in liquid/molten salts has been proposed and intensively investigated since 1980’s^14–26^. The major challenge for the molten salt technology, of which fluoride-based molten salt is dominant, is impurity control, due to the nature of fluoride-based molten salts and other multicomponent eutectic molten salts systems. Until now there has been no demonstration of a photovoltaic effect for a silicon film electrodeposited in fluoride-based molten salts. Therefore, chloride molten salt has been considered to be a promising alternative molten salt for silicon electrodeposition in recent years^25,27–29^.

In this work, we report the successful demonstration of a direct molten salt electrodeposition process of high purity (99.99989% (close to 6N)) crystalline silicon films in molten calcium chloride from abundant and inexpensive silicon dioxide. Calcium oxide is used as intermediate for the continuous ionization of silicon dioxide to form silicate ions in the molten salt. The doping type, either *p*-type or *n*-type, can be controlled by varying the dopants in the molten salt. Solar cell devices based on the as-prepared silicon films exhibit clear photovoltaic effects, with power conversion efficiency around 3.1%. This technique provides a promising approach for low-cost silicon solar cells production and potentially for high quality crystalline silicon film production for other applications.

## Results

### Design of electrodeposition of crystalline silicon films

Silicon dioxide is the primary source for silicon production. However, its solubility in chloride-based molten salts is generally low, inadequate for efficient electrodeposition^20–22,25^. Inspired by aluminum electrolysis in molten salt, efforts have been put into finding the right intermediate to facilitate the dissolution of silicon dioxide in chloride-based molten salts. Thanks to the considerable solubility of calcium oxide in molten calcium chloride and its reaction with silicon dioxide (Supplementary Note 1), the dissolution process from silicon dioxide to silicate ions is possible^28,29^, which offers access to the electrodeposition of high quality silicon films in molten calcium chloride.

**Fig. 1:**
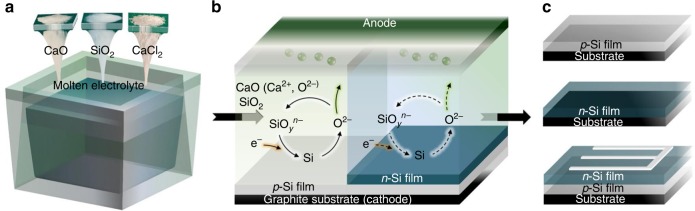
Schematic of the electrodeposition process for crystalline silicon films production. **a** The process starts from abundant and low-cost silicon dioxide in molten calcium chloride-calcium oxide electrolyte at 850 °C. **b** Direct electrodeposition of *p*-type, *n*-type and *p-n* junction silicon films through a cyclic reaction mechanism. **c** Schematics of the produced *p*-type, *n*-type silicon films and the possible solar cells based on the as prepared *p-n* junction silicon films.

As shown in Fig. 1, the only input materials for this molten salt electrodeposition of crystalline silicon films are abundant and low-cost silicon dioxide, calcium oxide and calcium chloride. Additional experimental details for the production of high-purity silicon films and reaction mechanisms can be found in Methods, Supplementary Tables 1 and 2, and Supplementary Figs. 1–6. Calcium oxide is added as an intermediate for continuing ionization of silicon dioxide to form silicate ions (expressed as SiO_*y*_^*n*−^, including SiO_3_^2−^, SiO_4_^4−^, etc.), which are then electrodeposited onto substrates to form crystalline silicon films. The general reactions for the electrodeposition process can be simply expressed as follows:

1 $$x\mathrm{SiO}_2 + y\mathrm{CaO}\left( {\mathrm{Ca}^{2 + },\mathrm{O}^{2-}} \right) \to \mathrm{Ca}_y\mathrm{Si}_x\mathrm{O}_{(2x + y)}\left( {y\mathrm{Ca}^{2 + },\mathrm{Si}_x\mathrm{O}_{(2x + y)}^{2y-}} \right)$$

2 $$\mathrm{Si}_x\mathrm{O}_{(2x + y)}^{2y-} + 4xe^- \to x\mathrm{Si} + \left( {2x + y} \right)\mathrm{O}^{2-}$$

As shown in reactions (1) and (2) and Fig. 1b, the electrodeposition route is a cyclic reaction process. By periodically feeding silicon dioxide into the molten salt, crystalline silicon films can be produced continuously, which makes this method suitable for large scale production. It has been proved that various dopants can be added into molten salt, such as boric anhydride or alumina for *p*-type and antimony oxide or calcium phosphate for *n*-type, to control the doping type of deposited silicon films. In addition, a proof-of-concept demonstration of *p*-*n* junction formation all by molten salt electrodeposition is shown in Fig. 1b, c and our recent work^28^. Therefore, crystalline silicon films with tunable film thickness and doping type can be facilely electrodeposited. Similar to the aluminum electrolysis process, this one-step molten salt electrodeposition process offers the potential to dramatically reduce the cost of silicon products.

### Characterization of crystalline silicon films

**Fig. 2:**
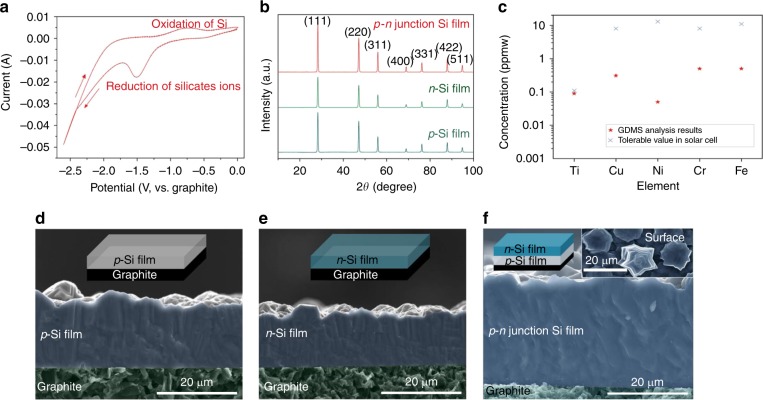
Characterizations of the electrodeposited crystalline silicon films in molten salt. **a** Cyclic voltammetry of the molten calcium chloride dissolved with silicon dioxide/calcium oxide at 850 °C with a scan rate of 50 mV s^−1^. **b** X-ray diffraction (XRD) patterns of the deposited *p*-type, *n*-type and *p-n* junction silicon films. **c** Glow discharge mass spectrometry (GDMS) analysis of the deposited silicon film, for selected impurities, of which have the most impact on solar cell efficiency. **d**–**f** Scanning electron microscopy (SEM) images of the **d** *p*-type, **e** *n*-type, and **f** *p*-*n* junction silicon films. The insets in (**d**–**f**) are the schematics showing the silicon films deposited on graphite substrate and the typical surface crystals (inset in (**f**)) of the deposited crystalline silicon films.

Silicon dioxide, calcium oxide and calcium chloride mixtures were first homogenized to form a molten electrolyte at 850 °C, and then silicate ions were gradually generated during the subsequent dissolution process. The silicate ions (denoted as SiO_*y*_^*n*−^, *e.g*. SiO_3_^2−^, SiO_4_^4−^, etc.) can be reduced to form silicon film on graphite substrates at approximately −1.5 V, as confirmed by CV results shown in Fig. 2a. By controlling the various dopants, either *p*-type and *n*-type silicon films can be produced. The CV results in Supplementary Fig. 7 reveal that the electrodeposition of *n*-type silicon film is also a simple reduction process and is similar to that of the *p*-type silicon films (Fig. 2a). We have demonstrated experimentally that crystalline silicon film can be deposited by using either constant potential/current density electrodeposition method or a pulse electrodeposition method. Pulse electrodeposition can yield dense and homogeneous silicon films formation, mainly due to the homogeneous silicate ion concentration at the cathode surface, which has been confirmed in our recent work^28,29^. The representative potential/current-time curves of the electrodeposition processes are shown in Supplementary Fig. 8. It is worth noting that calcium co-deposition would occur approximately at −2.7 V and calcium chloride would decompose at −3.2 V^29^. Therefore, the electrodeposition processes are all controlled at a potential less than −2.6 V. In addition, current density strongly influences the formation of dense films, with 15 to 20 mA cm^−2^ being the optimal current density. Higher or lower current densities will result in the formation of silicon powders and nanowires, respectively, as illustrated in Supplementary Fig. 9. By controlling current density, compact silicon films including *p-n* junction silicon films can be readily produced (Supplementary Figs. 10–12). The film thickness can be controlled in a range of about 5 µm to more than 60 µm, on various substrates, including graphite, silicon wafers and others^27–30^. The X-ray diffraction patterns, as shown in Fig. 2b, confirm the good crystallinity of as-prepared films. As studied in previous work^31^, Ti, Cu, Ni, Cr, and Fe are impurities having the most harmful impact on crystalline silicon solar cells, thus concentrations of these impurities were analyzed by glow discharge mass spectrometry (GDMS), as shown in Fig. 2c and Supplementary Fig. 13. It is clear to see that all the impurities levels in the electrodeposited silicon are below the tolerable threshold. The overall purity based on full spectrum GDMS analysis is calculated to be 99.99989% (close to 6N, solar grade). To the best of our knowledge, this is the highest purity yet reported for electrodeposited silicon in molten salts. For the doped silicon films, the dopant concentrations of P for *n*-type, and Al for *p*-type were characterized to be 3.5 and 10 ppm, respectively. We note that the impurity control is crucial for the electrodeposition of high-purity silicon films (Supplementary Figs. 3–5). Periodical pre-electrolysis process (about 120 h) was used to purify the molten salt to achieve an ultra-purified system, and then metallic impurities contained in the deposited silicon films can be strictly controlled at low level (e.g., magnesium less than 0.05 ppm, tungsten less than 0.05 ppm, sodium less than 0.05 ppm, calcium is about 5 ppm, etc.). In addition, to decrease the generation of carbon dioxide gas and influence the silicon films during electrodeposition, it has been experimentally demonstrated that the atmosphere needs to be strictly controlled by high-purity argon gas (flow rate of 50 to 100 mL min^−1^), current density and deposition potential also need to be remained at 10 to 20 mA cm^−2^ and less than −2.6 V, respectively. More details about the impurity control can be found in “Methods” and Supplementary Fig. 5.

The crystallinity, thickness and morphology of the deposited silicon films generally depend on the electrodeposition time, current density, and silicate ion concentration, as shown in Supplementary Figs. 14–18. In principle, *p*-type, *n*-type, and *p-n* junction silicon films with various thicknesses and surface morphologies can be produced by varying electrodeposition conditions, as preliminary confirmed in Fig. 2d–f and Supplementary Figs. 15–18. Energy dispersive spectroscopy (EDS) analysis further confirms that dense and uniform silicon films are deposited on graphite substrates, and no obvious boundary exists in the *p*-*n* junction silicon films, as shown in Supplementary Figs. 17f and 19. However, the growth rate of silicon films during the electrodeposition process is not constant. As shown in Supplementary Fig. 20, the growth rate is high within the first 4 h and then decreases over time. In addition, small amount of silicon powders would commonly generate on the surface of silicon films, which lower the film formation efficiency, and thus the current efficiency for the deposition of silicon films is hard to be accurately calculated. However, according to our previous work^28,29^ and based on this experimental observation, the current efficiency for the formation of silicon film is about 60 to 80%, depending on the growth rate of silicon film, which is not constant during the electrodeposition. The loss of current efficiency is mainly attributed to the formation of silicon powder on the film’s surface, which is expected can be further optimized by programming the deposition parameters.

### Device characterization and outlook

**Fig. 3:**
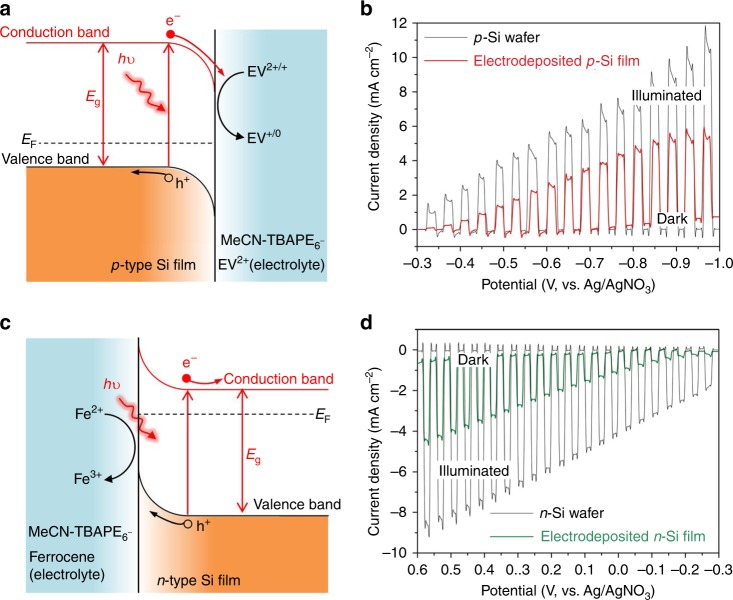
Photoelectrochemical characterization of the electrodeposited crystalline silicon films. **a** Schematic diagram of *p*-type silicon film/liquid junction with redox agent, where *E*_F_ and *E*_g_ are Fermi level and bandgap energy of silicon. **b** Photocurrent density-potential characteristics of the electrodeposited *p*-type silicon film and a commercial *p*-type silicon wafer in darkness and under illumination at 100 mW cm^−2^ with a scan rate of 10 mV s^−1^. **c** Schematic diagram of *n*-type silicon film/liquid junction with redox agent. **d** Photocurrent density-potential characteristics of the electrodeposited *n*-type silicon film and a commercial *n*-type silicon wafer in darkness and under illumination at 100 mW cm^−2^ with a scan rate of 10 mV s^−1^.

Characterization of a liquid-junction photoelectrochemical (PEC) cell enables rapid assessment of the quality of as prepared silicon films^32^. The fabrication and test of real solar cell devices will be discussed later. Here, electrodeposited *p*-type and *n*-type silicon films were prepared to form silicon/liquid junctions with redox agent and then characterized photoelectrochemically, as shown in Fig. 3. For comparison, commercial *p*-type and *n*-type silicon wafers were also characterized by PEC. The light is chopped on/off during the sweep and the photocurrent can be clearly observed. Figure 3b shows the photocurrent density of the as prepared *p*-type silicon film and a commercial *p*-type silicon wafer for reduction of ethyl viologen cations (EV^2+^). The photocurrent density of electrodeposited *p*-type silicon film is approximately 50% that of the commercial *p*-type silicon wafer. Figure 3d shows the photocurrent density of the as prepared *n*-type silicon film and commercial *n-*type silicon wafer for the oxidation of ferrocene. The photocurrent density of the electrodeposited *n*-type silicon film is about 40% that of the commercial *n*-type silicon wafer. For comparison, the PEC result of the electrodeposited silicon film without any dopant is shown in Supplementary Fig. 21. In addition, *p*-type and *n*-type silicon films electrodeposited under different conditions exhibit different PEC performances, as shown in Supplementary Figs. 22–24, suggesting the possibility to optimize the film quality by varying the electrodeposition conditions. The PEC performance of the deposited silicon film almost has no degradation after 6 months exposure in ambient (Supplementary Fig. 25).

**Fig. 4:**
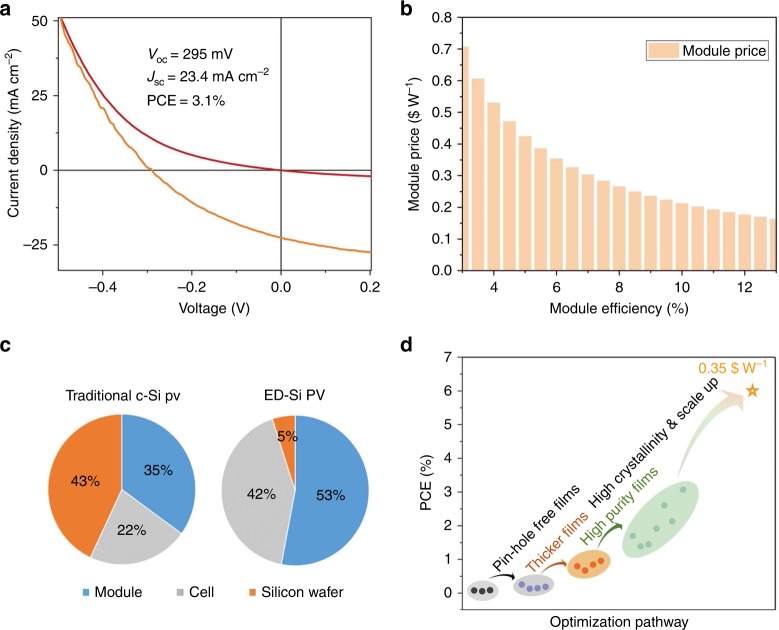
Solar cells efficiency characterization and cost analysis. **a** Solar cell devices based on the electrodeposited *p-*type silicon films. The devices are tested under dark (red line) and 100 mW cm^−2^ illumination, respectively. **b** Cost analysis based on molten salt electrodeposition technique. The manufacturing cost of solar cell module per watt is plotted *versus* module efficiency. **c** Comparison of cost breakdown for electrodeposited silicon photovoltaic (ED-Si PV) (10% power conversion efficiency (PCE)) and traditional crystalline silicon (c-Si) PV (21% PCE). **d** PCE versus the optimization pathway for this technology.

To compare with current silicon solar cell technology, solar cell devices were fabricated on the electrodeposited *p*-type silicon films as example. Device current density *versus* voltage is shown in Fig. 4a, with 295 mV open circuit potential (*V*_oc_), 23.4 mA cm^−2^ short circuit current (*J*_sc_) and 3.1% power conversion efficiency (PCE) being achieved. The cost benefit of silicon films by molten salt electrodeposition was further investigated by a detailed cost analysis^33–35^. More details of the cost analysis can be found in Supplementary Note 2 and Supplementary Table 3. Figure 4b is a brief summary of the dependence of total module cost ($ W_p_^−1^) on the module efficiency. It shows that a cell with only 6% and 10% PCE could enable 0.35 and 0.20 $ W_p_^−1^ total module cost, respectively, due to the significant reduction in the cost of the silicon wafer production, as presented in Fig. 4c. It is surprising to see that the fraction of silicon wafer cost can be reduced to 5% assuming a 10% PCE. The cell efficiencies have been enhanced steadily along with the improvement of film quality, including making uniform pin-hole free films, increasing the films thickness and reducing the impurities contents as shown in Fig. 4d and Supplementary Fig. 26.

## Discussion

In summary, we demonstrate a simple molten salt electrodeposition process for preparing crystalline silicon films for low-cost solar cells. *p*-type, *n*-type and *p-n* junction silicon films with tunable thicknesses can be directly produced from abundant and inexpensive silicon dioxide all in molten calcium chloride. The electrodeposited crystalline silicon films exhibit high-purity (99.99989% (close to 6N)) and clear photovoltaic effects with PCE as high as 3.1%. There is a large margin for improving the PCE with optimization of the electrodeposition process. Cost analysis further confirms that a module cost lower than 0.20 $ W_p_^−1^ can be achieved with PCE higher than 10%, making this technology promising for low-cost silicon solar cells.

## Methods

### Materials

Silicon dioxide (SiO_2_, Sigma-Aldrich, nanopowder, 10 to 20 nm, with purity of 99.5%, trace metals basis), calcium oxide (CaO, Sigma-Aldrich, with purity of 99.9%, trace metals basis) and calcium chloride (CaCl_2_, Sigma-Aldrich, ACS reagent, with purity of 99%, St. Louis, MO. Ba less than 0.005%, Fe less than 0.001%, K less than 0.01%, Mg less than 0.005%, NH_4_^+^ less than 0.005%, Na less than 0.02%, Sr less than 0.01%, heavy metals less than 5 ppm) were used to form a molten electrolyte for electrodeposition. Antimony oxide (Sb_2_O_3_, Sigma-Aldrich, with purity of 99.999%) and calcium phosphate (Ca_3_(PO_4_)_2_, Sigma-Aldrich, 4 μm, total heavy metals: less than 20 ppm) powders were used to provide antimony and phosphorus as dopants for *n*-type silicon film, respectively. Alumina (Al_2_O_3_, Sigma-Aldrich, with purity higher than 99.9%) and boric anhydride (B_2_O_3_, Sigma-Aldrich, with purity of 99.999%) were used to provide aluminum and boron as dopants for *p*-type silicon film, respectively. High-purity quartz crucible (Technical Glass Products, O.D. 40 × I.D. 37 × height 180 mm, Painesville, OH. Al: 0.5 ppm, B less than 0.2 ppm, Ca: 0.4 ppm, Cu less than 0.05 ppm, Cr less than 0.05 ppm, Fe: 0.2 ppm, K: 0.6 ppm, Li: 0.6 ppm, Mg: 0.1 ppm, Mn less than 0.05 ppm, Na: 0.7 ppm, Ni less than 0.1 ppm, P less than 0.2 ppm, Sb less than 0.003 ppm, Ti: 1.1 ppm, Zr: 0.8 ppm) was used as electrolytic cell. POCO graphite plate (AXF-5Q, Entegris POCO, Decatur, TX, US; 75 mm in length, 6–20 mm in width, and 1 mm in thickness) was used as substrate for the electrodeposition of silicon. Graphite rod (Alfa Aesar, with purity of 99.995%, diameter 6 mm, Haverhill, MA, US) or the POCO graphite plate was used as anode. Tungsten wires (Alfa Aesar, with purity of 99.9%, diameter 1 mm) were used as the electrode leads and protected by quartz tubes (Technical Glass Products, 3 mm, 6 mm and 10 mm in diameter).

### Electrodeposition of crystalline silicon films

Crystalline silicon films were electrodeposited directly from the molten electrolyte containing silicon dioxide/calcium oxide/calcium chloride. In a typical experiment, 100 g calcium chloride, 2.2 g silicon dioxide, 2.0 g calcium oxide were mixed together and homogenized at 850 °C to form a molten electrolyte in a quartz crucible, which was placed in a one-end closed quartz tube and heated in a furnace. The entire reaction system was sealed and high-purity argon gas (99.99%) was purged into the tube to form argon gas atmosphere during the electrodeposition process. The electrolytic cell was kept at 850 °C for 24–48 h to form sufficient silicates ions concentration, and then pre-electrolysis was periodically performed between two graphite rods at 2.5 to 2.8 V for ~120 h to remove the possible impurities. We note that the periodical pre-electrolysis process is the key step for the production of high-purity silicon films. To achieve an ultra-purified molten salt system, more than 120 h periodical pre-electrolysis process is needed. In addition, the anode and cathode are also important for the electrodeposition process. We use high-purity carbon/graphite rod as contact in high temperature zone to avoid any metallic wire/element close/around molten salt, the details about the fabricated electrodes are shown in Supplementary Figs. 3 and 4. And the complete time-related electrodeposition processes for high-purity silicon films including pre-electrolysis, dissolution, and electrodeposition processes are shown in Supplementary Fig. 5. To provide enough silicates ions concentration for the electrodeposition process, high-purity silicon dioxide and dopant compounds can be periodically added into the purified molten salt system. After the sufficient pre-electrolysis and dissolution processes, cyclic voltammetry (CV) experiment was firstly carried out in the molten calcium chloride electrolyte dissolved with silicon dioxide/calcium oxide at 850 °C with a scan rate of 50 mV s^−1^, more details about the CV experiment can be found in our previous work^28,29^. Then, the electrodeposition experiment was carried out at 10–20 mA cm^−2^ in the two-electrode manner with a graphite plate as substrate and a graphite rod/plate as anode in an argon gas atmosphere. *p*-type, *n*-type, and *p-n* junction silicon films can be electrodeposited by controlling the electrodeposition and doping processes. For the electrodeposition of *p*-type silicon film, alumina or boric anhydride powders can be added into the molten electrolyte to provide aluminum or boron as dopant. For the electrodeposition of *n*-type silicon film, calcium phosphate or antimony oxide powders can be added into the molten electrolyte to provide phosphorus or antimony as dopant. For the electrodeposition of *p-n* junction silicon film, the electrodeposition process was divided into two periods, i.e., the first period for the electrodeposition of *p*-type silicon film and the second period for the electrodeposition of *n*-type silicon film on the *p*-type silicon film. About the doping mechanism, dissolution-electrodeposition reaction processes may occur during silicon electrodeposition, as shown in Supplementary Table 2 and discussed in our recent work^28,29^. After the electrodeposition process, the electrodeposited silicon film was removed slowly from molten electrolyte and cool down in an argon gas atmosphere. Then the film was taken out and washed with deionized water and ethanol as well as dried at 100 °C. More experimental details can be found in our recent work^28,29^. We note that the graphite-based anode may raise the concerns over carbon dioxide generations and emissions. For molten salt electrolysis in calcium chloride with carbon anode, it is reported that the generated carbon dioxide gas might be partially dissolved in molten salt and contaminates the cathode^24,25^. However, in this work, the electrodeposition process was carried out at low current/current density and the atmosphere was strictly protected by high-purity argon gas (at a flow rate of 50–100 mL min^−1^), so the generated anodic gas is mainly carbon monoxide (~97%) and can be purged out immediately. Besides, the calcium oxide concentration in molten calcium chloride also has influence on the formation of carbon monoxide gas during electrodeposition^36^, thus the calcium oxide addition in molten calcium chloride needs to be strictly controlled at about 2.0 wt%. In this work, it has been experimentally demonstrated that the molten salt system can keep clean even after running for 20 to 30 days, however, more details about the influence of carbon on silicon film need further investigation. In addition, the graphite substrate may also influence the performance of the produced silicon films, new substrates and inert anode for the electrodeposition process are currently being investigated. Accordingly, a considerable improvement of the performance of the deposited silicon films would be expected based on the further optimization.

### Silicon films characterization

The deposited silicon films were characterized by using scanning electron microscopy (SEM, Quanta 650 FEG, FEI Inc., Hillsboro, OR) and energy dispersive spectroscopy (EDS, XFlash Detector 5010, Bruker, Fitchburg, WI). The impurity concentration of the silicon film was analyzed by glow discharge mass spectrometry (GDMS, VG 9000, Thermo Fisher Scientific Inc., Waltham, MA, USA). X-ray diffraction spectroscopy (XRD, Philips X-ray diffractometer equipped with Cu Kα radiation) was also used to analyze the produced silicon films. The electrodeposited silicon films were tested as photoelectrodes for the PEC measurement. For the *p*-type silicon film, the PEC test was carried out in an argon gas-purged acetonitrile (CH_3_CN, 99%, Extra-dry, Acros, Fair Lawn, NJ) solution containing 0.1 M tetrabutylammonium hexafluorophosphate (TBAPF_6_, with purity higher than 99.9%, Fluka, Allentown, PA) as supporting electrolyte and 0.05 M ethyl viologen diperchlorate (EV(ClO_4_)_2_, Sigma-Aldrich, with purity of 98%) as the redox agent. For the *n*-type silicon film, the PEC test was performed in an argon gas-purged CH_3_CN solution containing 0.1 M TBAPF_6_ as supporting electrolyte and 0.05 M Ferrocene (Fe(C_5_H_5_)_2_, Sigma-Aldrich, St. Louis, MO) as the redox reagent. The PEC properties under UV-visible light illumination by a xenon lamp at 100 mW cm^−2^ were tested and compared with commercial *p*-type and *n*-type silicon wafers (University Wafers, 5 to 10 ohm-cm, (100), boron-doped (*p*-silicon wafer), phosphorus-doped (*n*-silicon wafer), Boston, MA, US).

### Device fabrications and characterization

The electrodeposited silicon films were first mechanically polished and rinsed. Then the shallow *p*-*n* junction was made by spin-on-dopant, including spin-coating and rapid thermal annealing for dopant activation (950 °C, 60 s). The top contact patterns were then made by lithography and metallization. The I-V characterization of solar cell devices was performed using a B1500A Semiconductor Device Analyzer (Agilent Technologies) and Summit 11000 AP probe station (Cascade Microtech). Solar simulator (Newport) with AM 1.5G filter, calibrated to 100 mW cm^−2^, was used as light source.

### Cost analysis

The cost analysis model is based on sum of costs of each fabrication steps. The cost of electrodeposition silicon films is analyzed by ownership model includes costs associated with materials, labor fees, depreciation and maintenance of equipment and facilities. Detailed data are collected from industry members, vendors and official reports. For cell fabrication processes and module assembling, costs are used based on state-of-art technologies, collected from various resources, including publicly available databases and official reports. More details can be found in Supplementary Note 2.

## Supplementary information

Supplementary Information

## Data Availability

The data that support the findings of this study are available from the corresponding author upon reasonable request.
