# Supplementary material for: Electrodeposition of crystalline silicon films from silicon dioxide for low-cost photovoltaic applications
Source: Nat Commun. 2019 Dec 18;10:5772. doi: 10.1038/s41467-019-13065-w (PMC6920409; doi:10.1038/s41467-019-13065-w)
Supplement: Supplementary file 1 — Supplementary Information [file 41467_2019_13065_MOESM1_ESM.pdf]

# Supplementary Information

## Electrodeposition of crystalline silicon films from silicon dioxide for low-cost photovoltaic applications

**Zou *et al.***

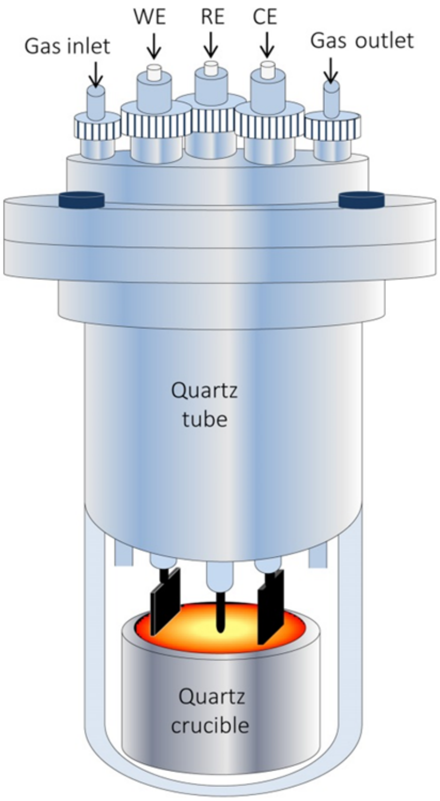


**Supplementary Figure 1 | Schematic of experimental setup.**

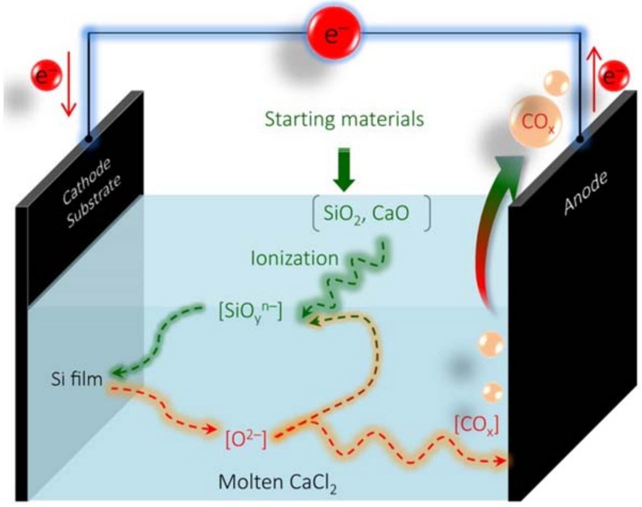


**Supplementary Figure 2 | Schematic of the electrolytic cell proposed for the molten salt electrodeposition of crystalline silicon films.**

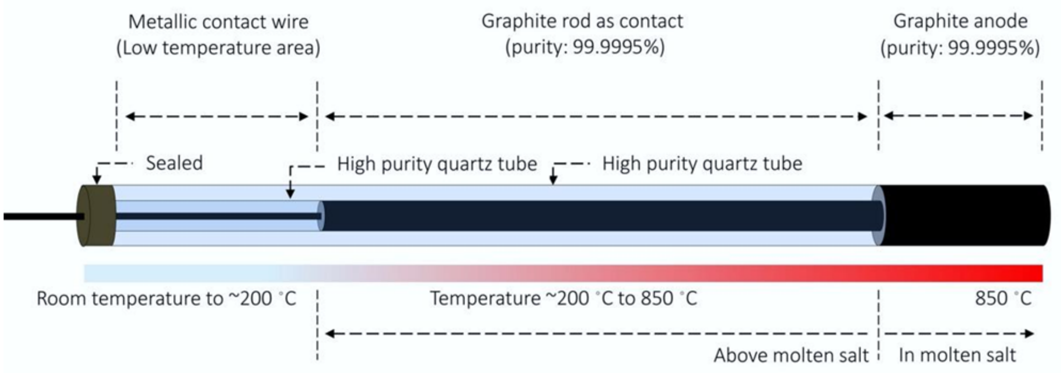


**Supplementary Figure 3 | Schematic of the fabricated graphite anode (rod or plate) for the high purity silicon film deposition.**

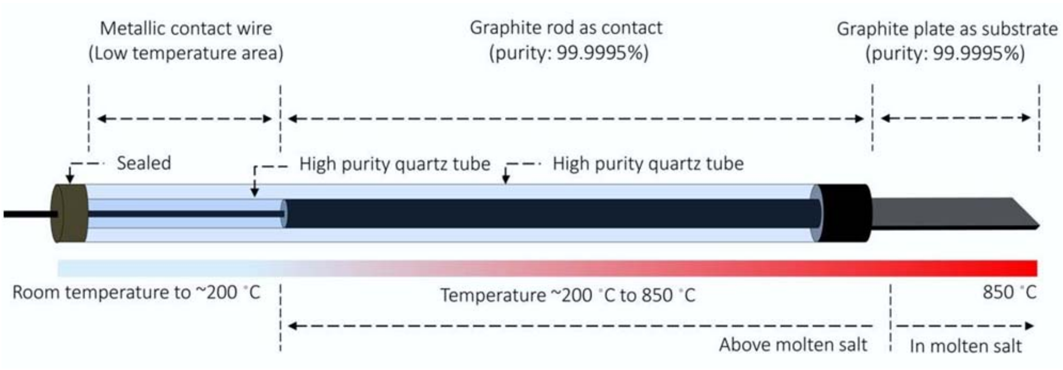


**Supplementary Figure 4 | Schematic of the fabricated cathode graphite substrate for the high purity silicon film deposition.**

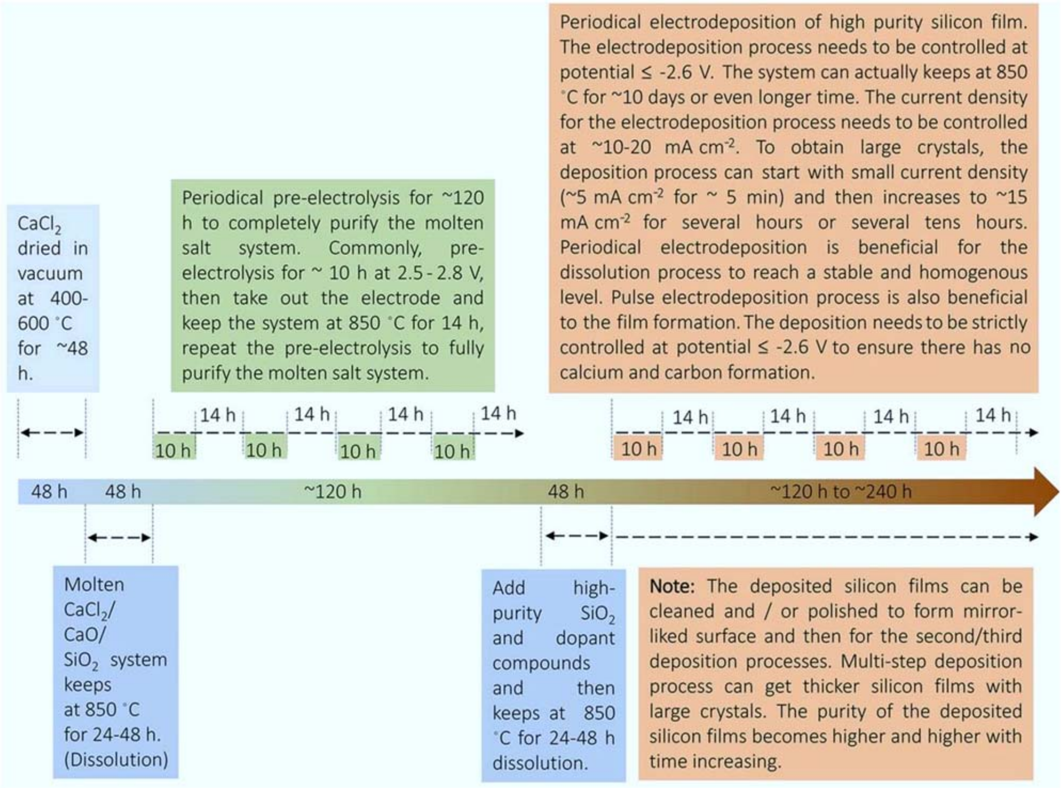


**Supplementary Figure 5 | Schematic showing the complete time-related process for the electrodeposition of high purity silicon films.**

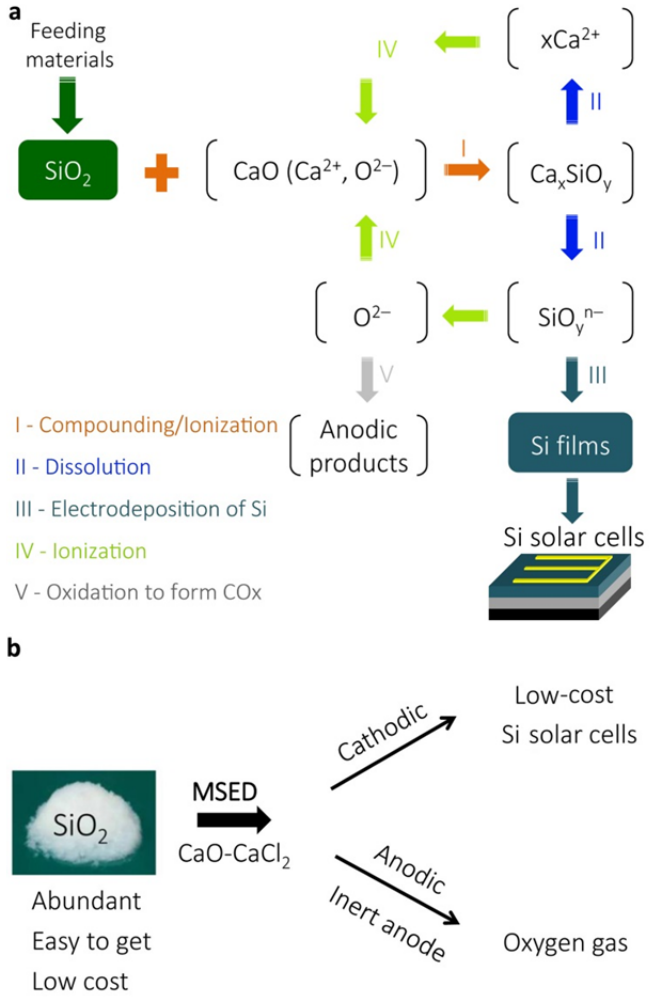


**Supplementary Figure 6 | Schematic of the simple strategy for the molten salt electrodeposition (MSED) of crystalline silicon films from silicon dioxide in molten calcium chloride. a**, The cyclic reaction route showing the reaction mechanism. **b**, The possible promising design for a sustainable silicon solar cells production process.

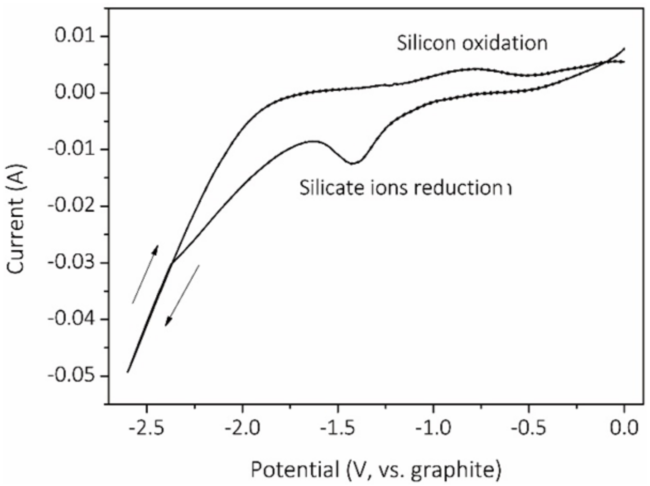


**Supplementary Figure 7 | Cyclic voltammetry (CV) analysis.** CV curve of the molten calcium chloride dissolved with silicon dioxide and calcium oxide at 850 ºC with the addition of *n*-type dopant compounds for the electrodeposition of *n*-type silicon films (scan rate is 50 mV $s^{-1}$).

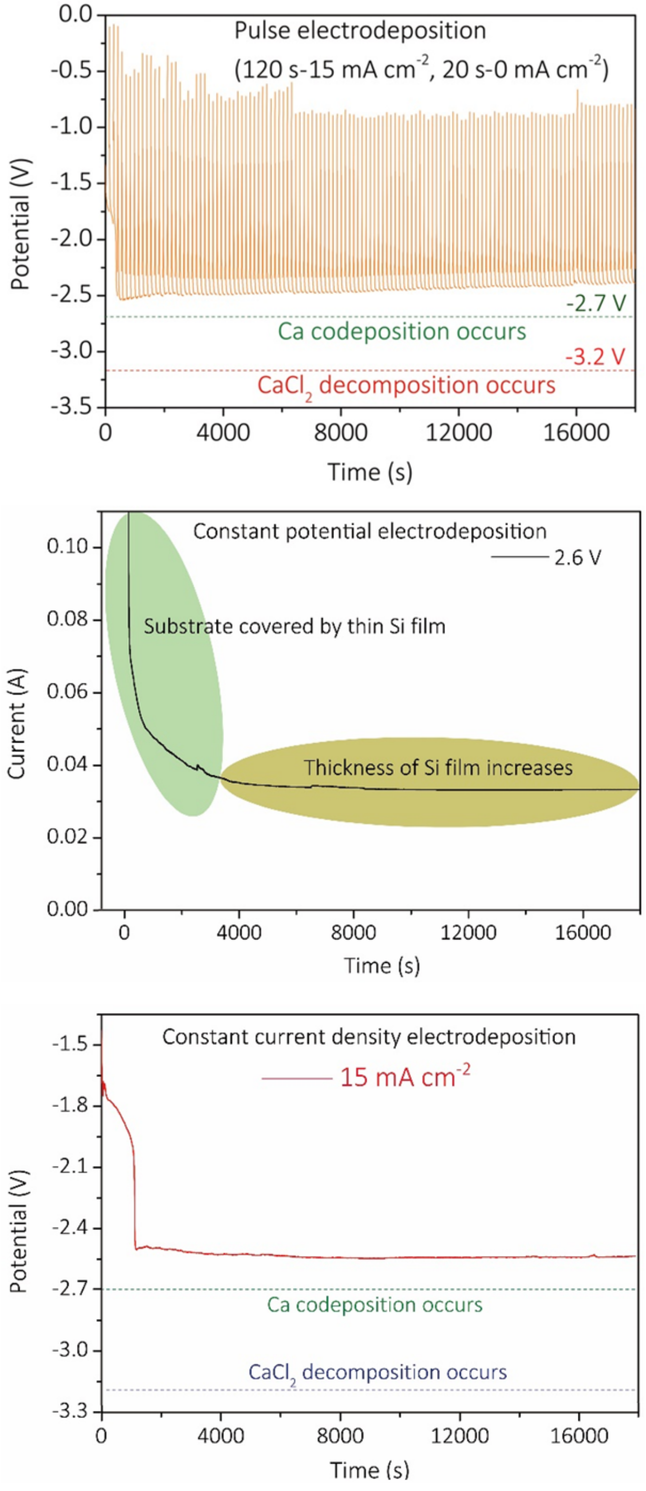


**Supplementary Figure 8 | Typical current/potential-time curves of the pulse electrodeposition, constant potential/current density electrodeposition processes for crystalline silicon films at 850 ºC.**

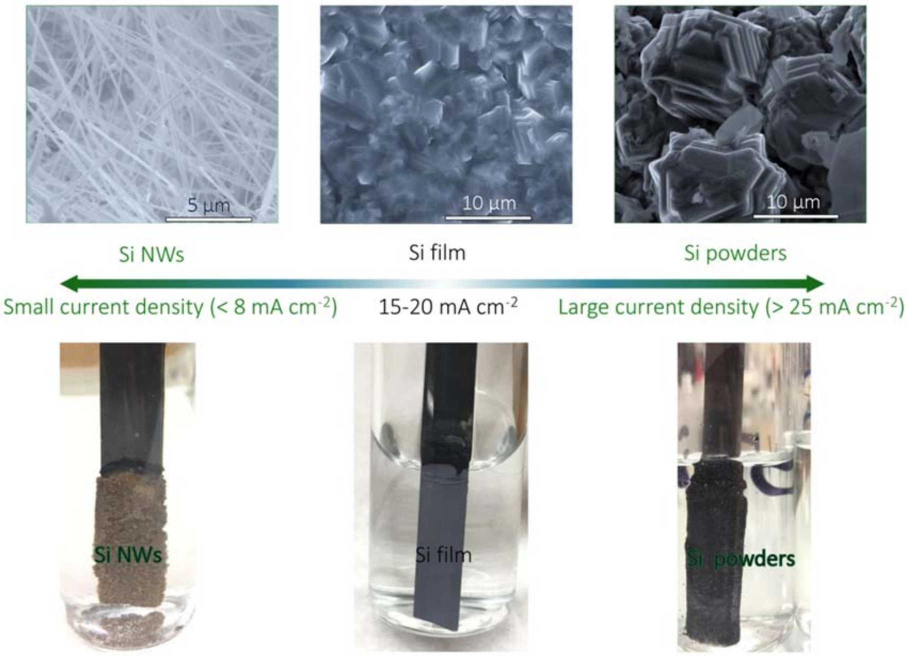


**Supplementary Figure 9 | Typical photos and scanning electron microscopy (SEM) images of the silicon deposited at different current densities at 850 ºC.** 15 to 20 mA $cm^{-2}$ is the optimum current density for the formation of dense silicon film, and lower current density (lower than 8 mA $cm^{-2}$) generally leads to the formation of silicon nanowires, high current density (higher than 25 mA $cm^{-2}$) commonly leads to the formation of silicon powders.

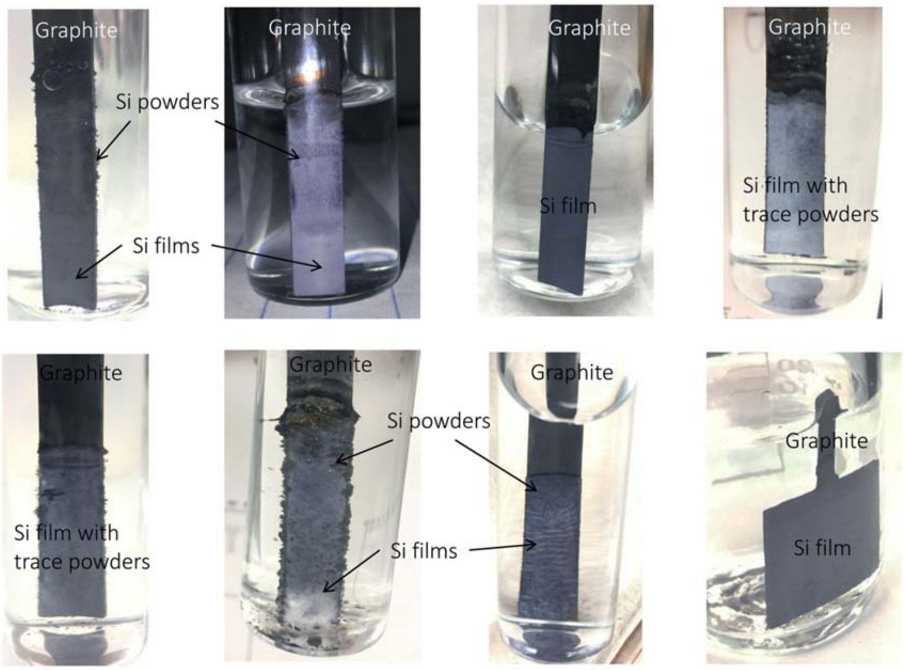


**Supplementary Figure 10 | Photos of the silicon films deposited at 15 to 20 mA cm$^{-2}$**. The small amount of silicon powders on the surface of films can be easily washed through water.

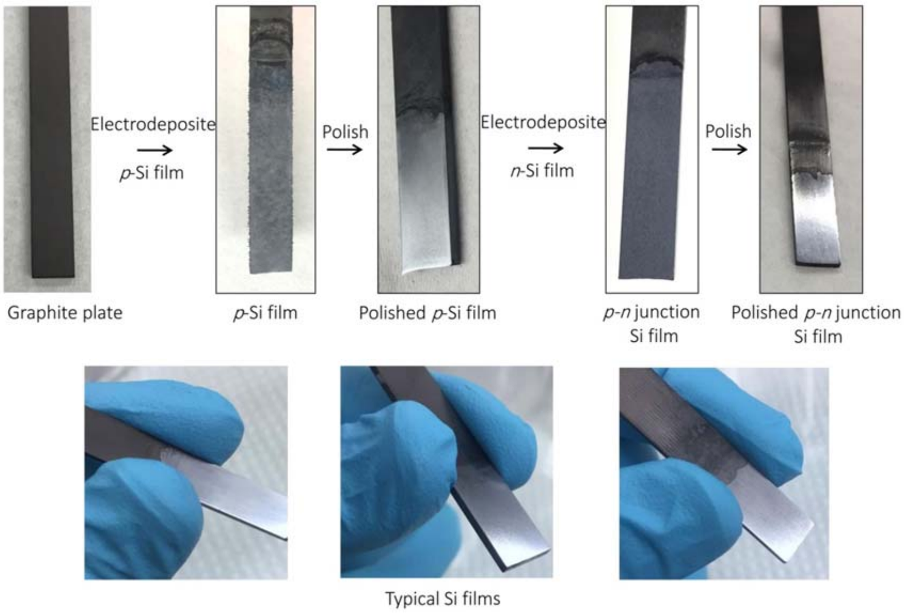


**Supplementary Figure 11 | Typical photos of the electrodeposited silicon films showing the two-period electrodeposition process for the deposition of *p*-*n* junction silicon films.** The surface morphology of the deposited crystalline silicon film can be controlled to form smooth surface, which can be directly used for the deposition of *n*-type silicon film without polishing.

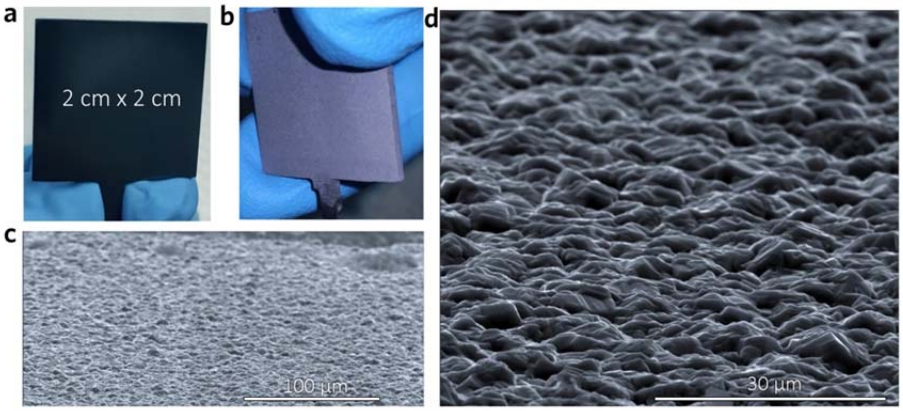


**Supplementary Figure 12 | Photos and SEM images of the crystalline silicon films deposited on graphite substrate.** **a** and **b**, Photos of the graphite substrate before and after being deposited with crystalline silicon film. **c** and **d**, Surface's morphology of the deposited crystalline silicon film.

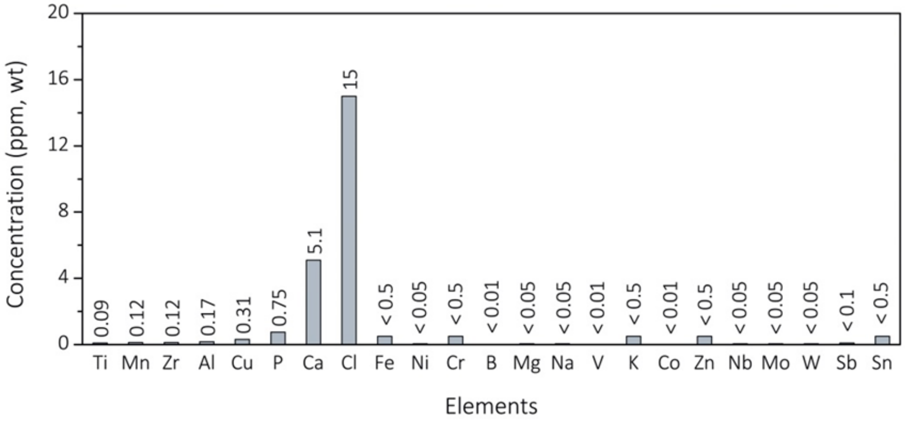


**Supplementary Figure 13 | The impurity elements of a typical deposited silicon films determined by glow discharge mass spectrometry (GDMS) analysis.** Ca and Cl elements come from the residual calcium chloride, which can be further removed through procedure optimization. C, H, and O are not determined, C element is hard to be accurately analyzed because of the substrate is also graphite; impurities not listed are below detection limit.

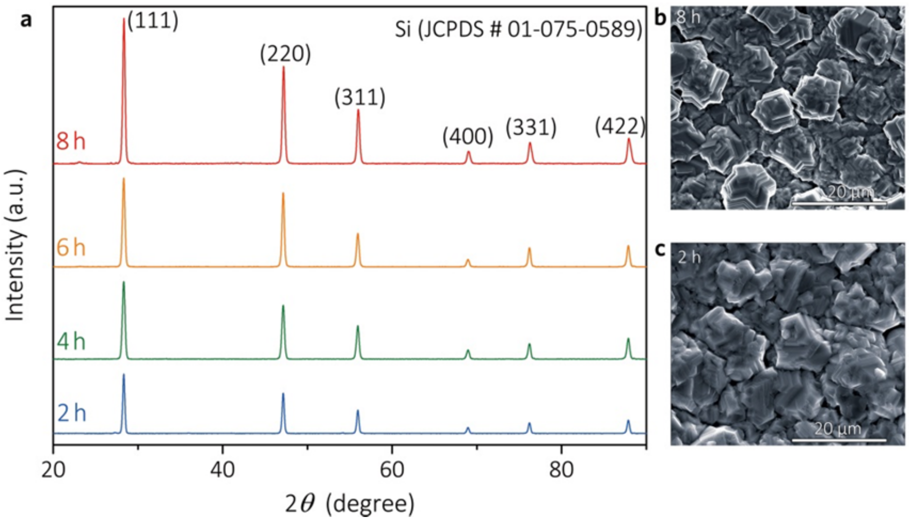


**Supplementary Figure 14 | Characterization of the deposited crystalline silicon films.** **a**, X-ray diffraction (XRD) patterns of the silicon films obtained from different deposition times at 850 ºC. **b**, SEM image of the surface of the silicon film deposited for 8 h. **c**, SEM image of the surface of the silicon film deposited for 2 h.

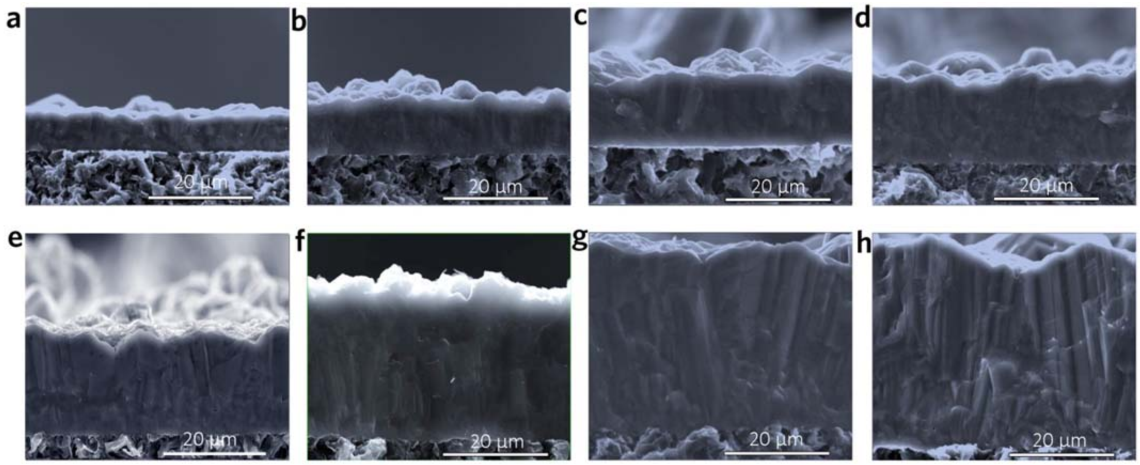


**Supplementary Figure 15 | a-h, SEM images of the cross sections of the *p*-type silicon films with different thicknesses.** These films were deposited on graphite substrate at 850 ºC with current density of 15 to 20 mA $cm^{-2}$.

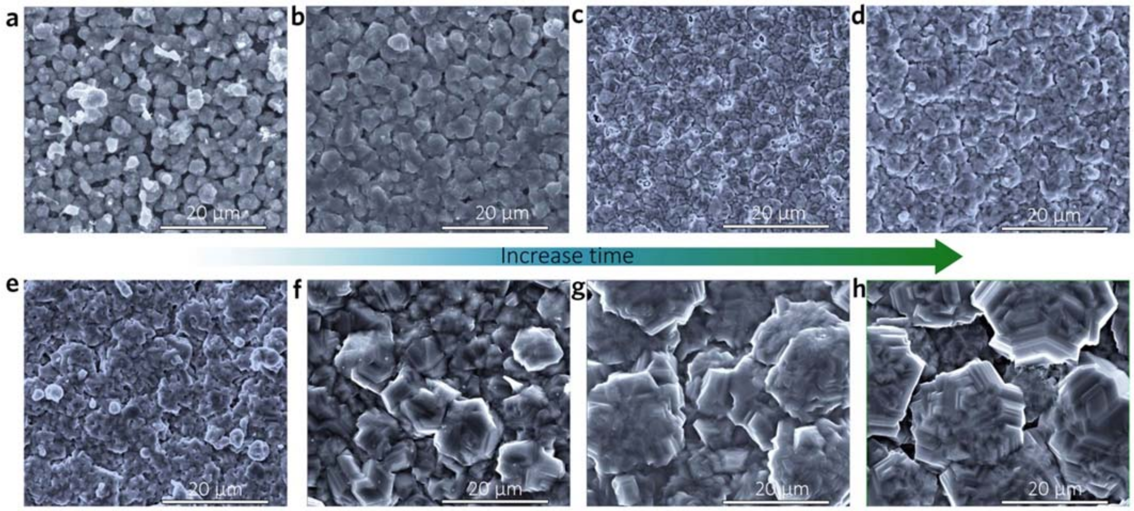


**Supplementary Figure 16 | a-h, SEM images of the surfaces of the *p*-type silicon films with different morphologies.** These films were deposited on graphite substrate at 850 ºC with current density of 15 to 20 mA cm$^{-2}$. Obviously, the small silicon crystallites gradually grow up to form large crystallites. It should be noted that the morphology of the surface of silicon films can be controlled by adjusting current density, *etc*. Dense silicon films with relatively smooth surfaces can be obtained through molten salt electrodeposition.

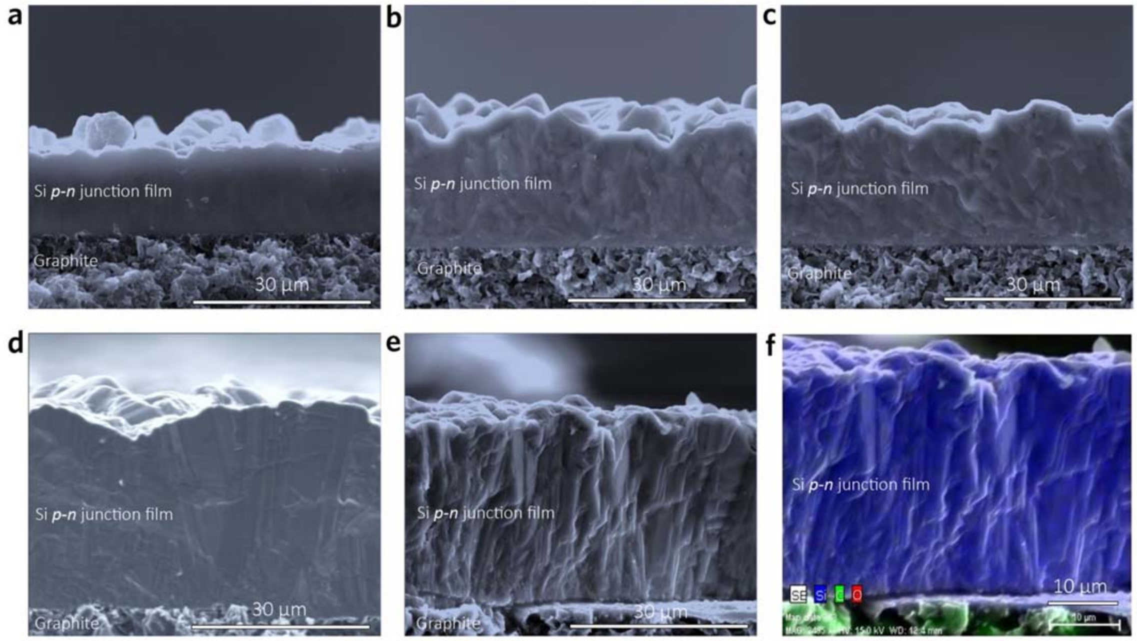


**Supplementary Figure 17 | SEM images and energy dispersive spectroscopy (EDS) map of the *p*-*n* junction silicon films. a-e,** SEM images of the cross sections of the *p*-*n* junction silicon films with different thicknesses. **f**, EDS map of the *p*-*n* junction silicon film. These films were deposited on graphite substrate at 850 ºC with current density of 15 to 20 mA $cm^{-2}$.

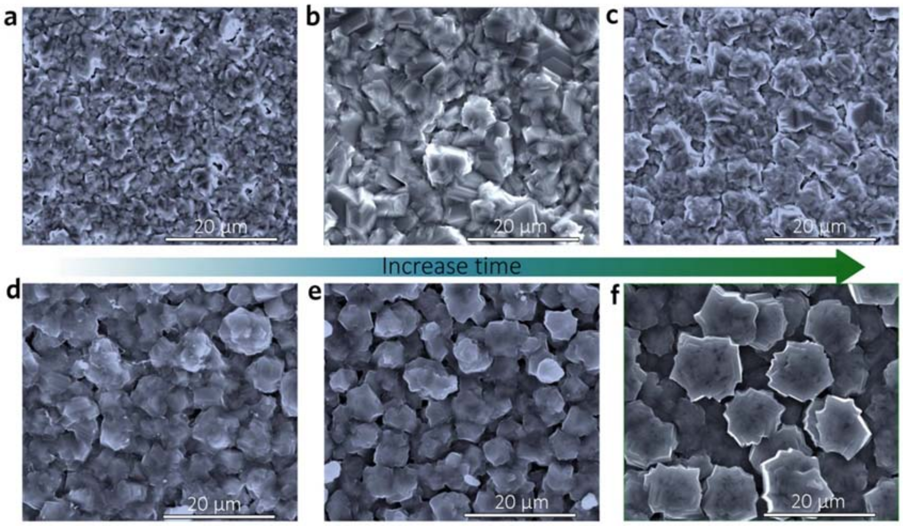


**Supplementary Figure 18 | a-h, SEM images of the surfaces of the *p-n* junction silicon films with different surface morphologies.** These films were deposited on graphite substrate at 850 ºC with current density of 15 to 20 mA $cm^{-2}$. Generally, the small silicon crystallites gradually grow up to form large crystallites.

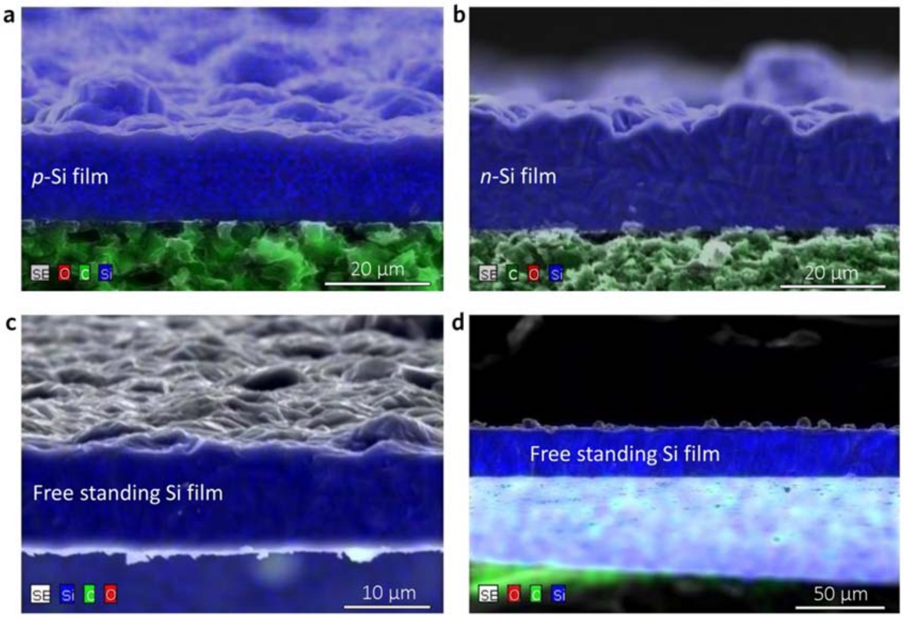


**Supplementary Figure 19 | EDS maps of the crystalline silicon films. a** and **b**, EDS maps of the *p*-type and *n*-type silicon films deposited on graphite substrate. **c** and **d**, EDS maps of the free standing silicon films detached from graphite substrate. Free standing silicon films can be easily obtained by oxidation of the graphite substrate in air at 600 to 650 °C.

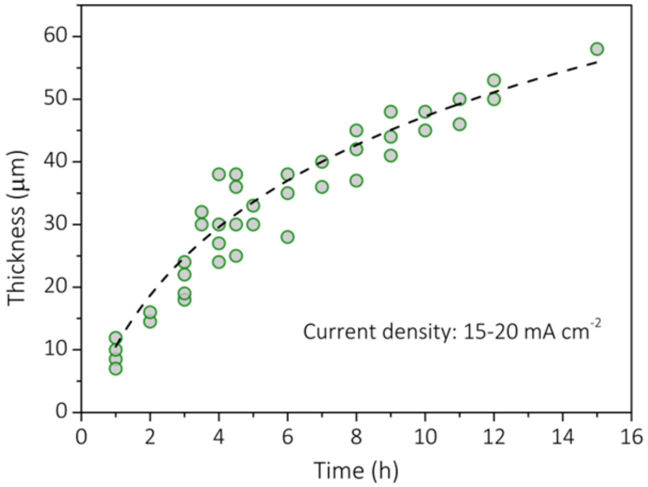


**Supplementary Figure 20 | Relationship plot of the thickness of silicon film with electrodeposition time.**

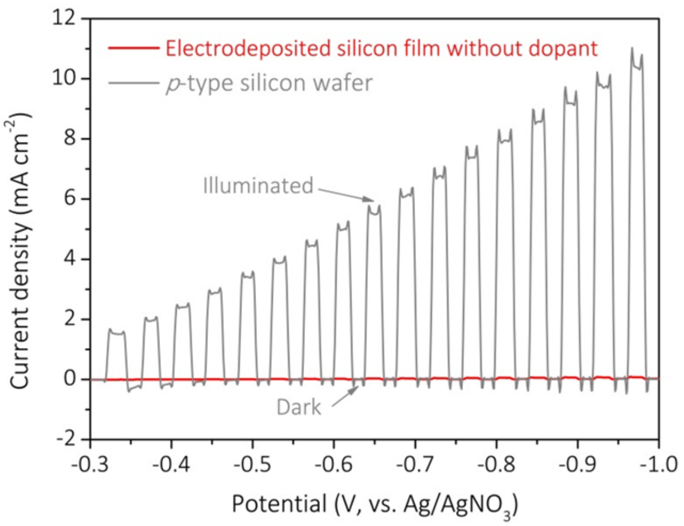


**Supplementary Figure 21 | Comparison of the photoelectrochemical (PEC) result of *p*-type silicon wafer and the electrodeposited high purity silicon film without any dopant.** The silicon film without any dopant was electrodeposited by using high purity quartz crucible, and periodical pre-electrolysis process was firstly carried out to remove the possible impurity before electrodeposition of high purity silicon film without any dopant.

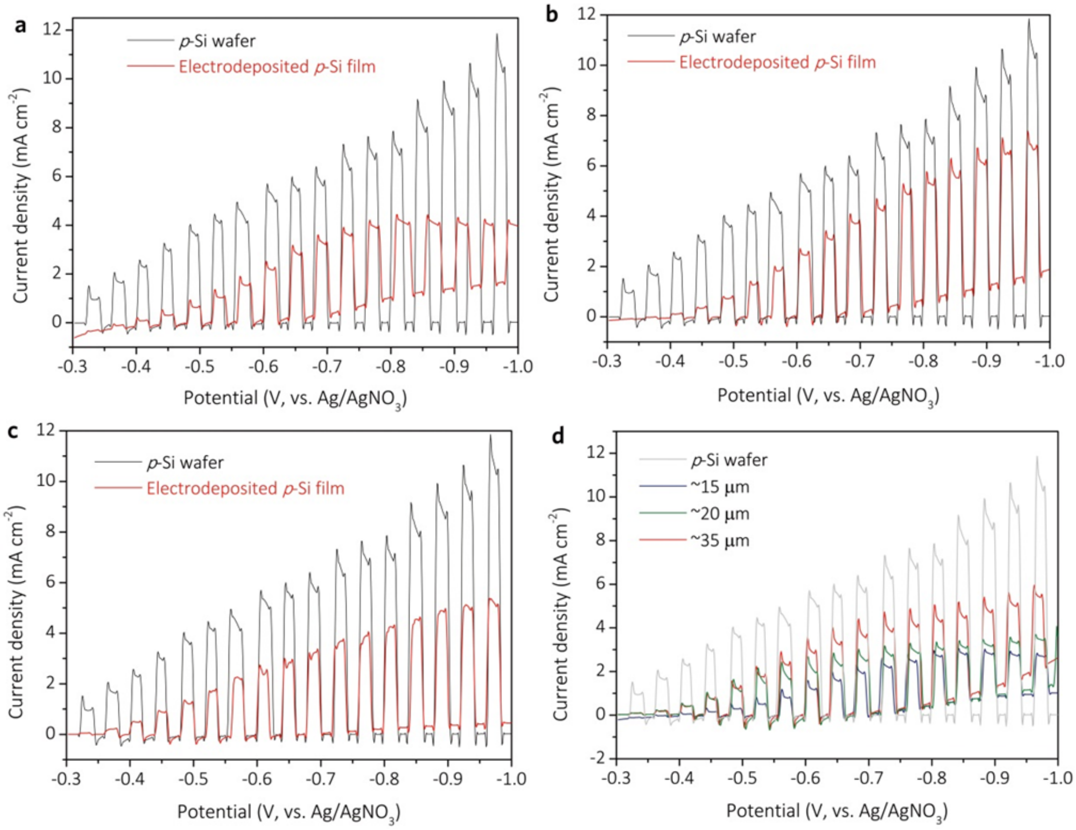


**Supplementary Figure 22 | PEC characterization of the *p*-type silicon films. a-c**, Typical PEC results of the *p*-type silicon films deposited from $SiO_2$/CaO/$CaCl_2$ electrolyte compared with *p*-type silicon wafer. **a**, Silicon film with small crystallites (5 to 8 μm). **b**, Silicon film with medium-size crystallites (10 to 15 μm). **c**, Silicon film with large crystallites (20 to 25 μm). **d**, PEC results of the deposited *p*-type silicon films with different thicknesses.

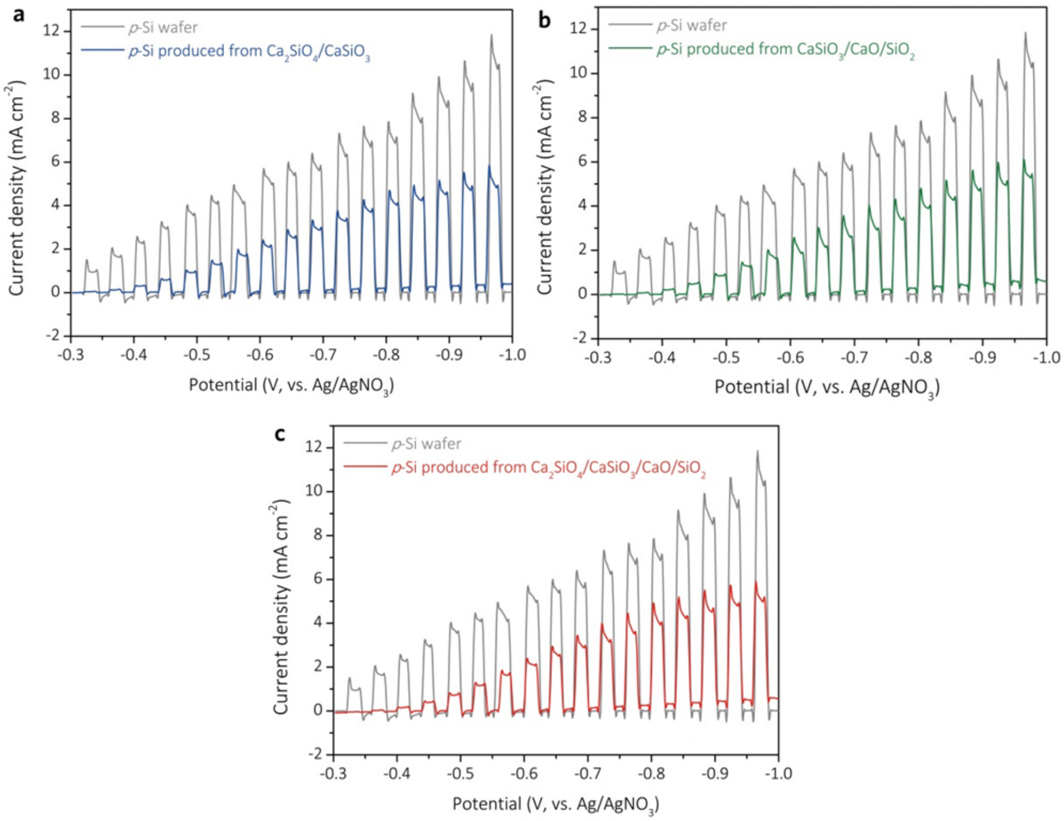


**Supplementary Figure 23 | Typical PEC results of the *p*-type silicon films. a-c,** Typical PEC results of the *p*-type silicon films deposited from different precursors. **a**, Silicon film deposited from $Ca_2SiO_4/CaSiO_3$. **b**, Silicon film deposited from $CaSiO_3/CaO/SiO_2$. **c**, Silicon film deposited from $Ca_2SiO_4/CaSiO_3/CaO/SiO_2$. The calcium silicates $Ca_2SiO_4$ and $CaSiO_3$ were synthesized from CaO and $SiO_2$. Generally, these silicon films show good photoresponse, the photocurrent densities of these silicon films are about 50% that of the commercial *p*-type silicon wafer.

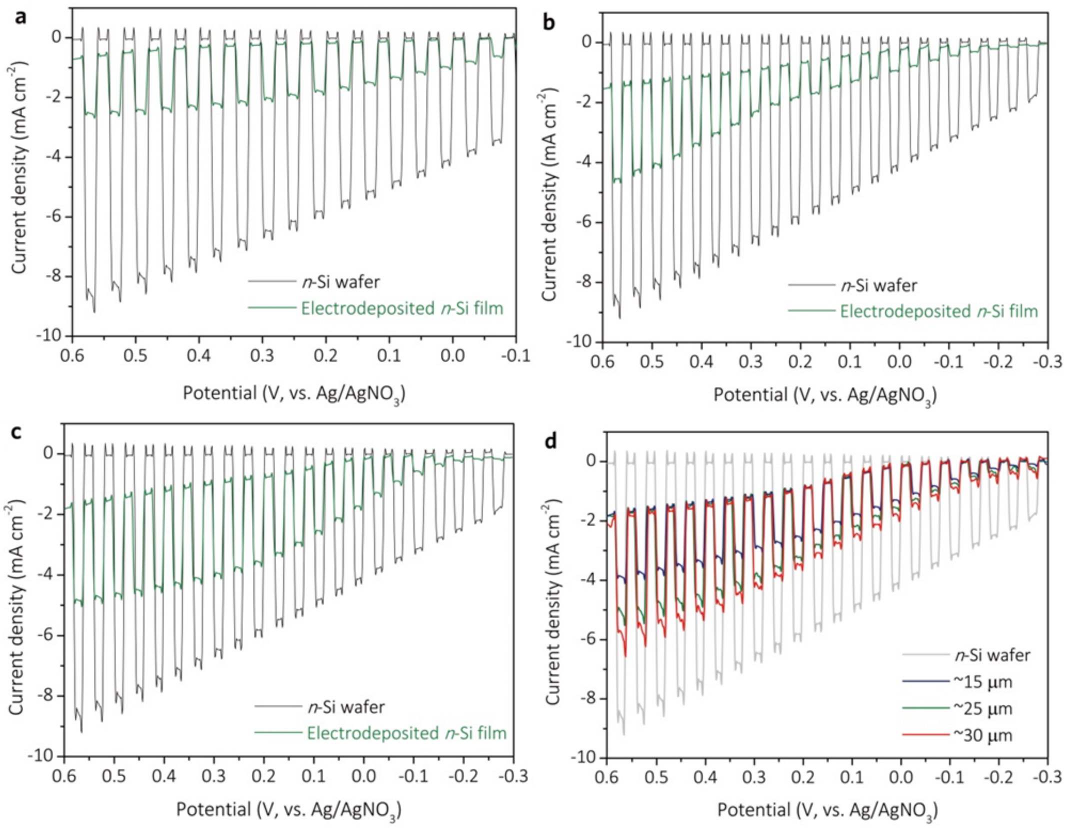


**Supplementary Figure 24 | Typical PEC results of the *n*-type silicon films. a-c**, Typical PEC results of the *n*-type silicon films deposited from $SiO_2$/CaO/$CaCl_2$ electrolyte compared with *n*-type silicon wafer. **a**, Silicon film with small crystallites (5 to 8 μm). **b**, Silicon film with medium-size crystallites (10 to 15 μm). **c**, Silicon film with large crystallites (20 to 25 μm). **d**, PEC results of the deposited *n*-type silicon films with different thicknesses.

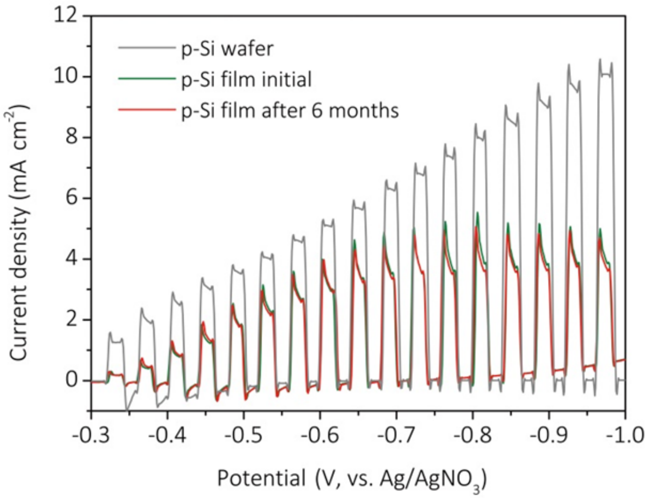


**Supplementary Figure 25 | Typical PEC of the *p*-type silicon film after 6 months and compare to its initial PEC behavior.**

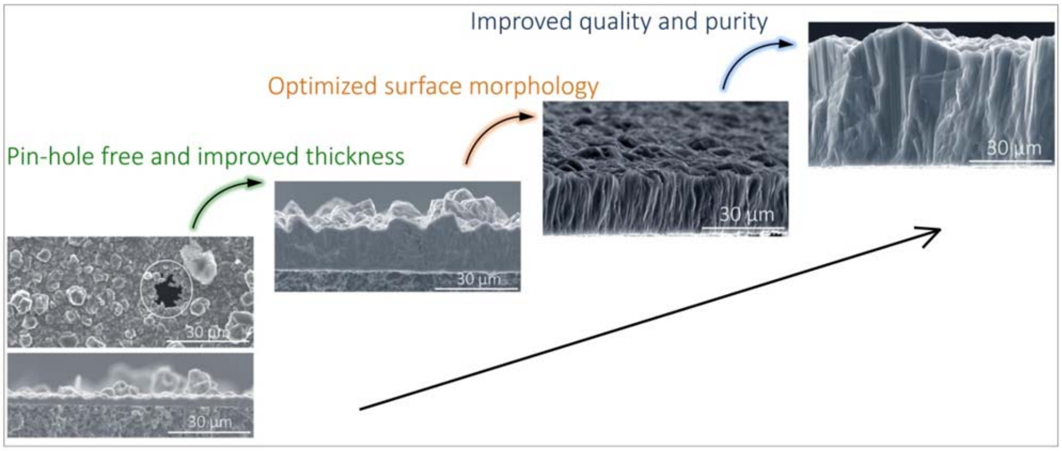


**Supplementary Figure 26 | SEM images of typical silicon films showing the optimization process.**

**Supplementary Table 1 |** Summary of the different electrodeposition conditions used for the production of crystalline silicon films in this work.*

| **Electrolyte composition** | **Current density ($mA\ cm^{-2}$)** | **Thickness (μm) (Time: 1 to 15 h)** | **Silicon type** | **Dopant (compounds)** |
|---|---|---|---|---|
| $SiO_2$/CaO/$CaCl_2$ | 15 to 20 | 5 to 60 | *p*-Si, *n*-Si, *p*-*n* junction Si films | Al ($Al_2O_3$) or B ($B_2O_3$) for *p*-Si films;<br>Sb ($Sb_2O_3$) or P ($Ca_3(PO_4)_2$) for *n*-Si films |
| $CaSiO_3$/$CaCl_2$ | 15 to 20 | 5 to 30 | *p*-Si, *n*-Si films | |
| $SiO_2$/$CaSiO_3$/CaO/$CaCl_2$ | 10 to 20 | 5 to more than 60 | *p*-Si films | |
| $CaSiO_3$/$Ca_2SiO_4$/$CaCl_2$ | 15 to 20 | 5 to 40 | *p*-Si films | |
| $SiO_2$/$CaSiO_3$/$Ca_2SiO_4$/CaO/$CaCl_2$ | 10 to 20 | 5 to more than 60 | *p*-Si films | |

***Note:** For comparison, we also tried to synthesize calcium silicates ($Ca_2SiO_4$/$CaSiO_3$) from CaO and $SiO_2$ and then used as precursors for the electrodeposition of crystalline silicon films in molten salts, which suggests that the reaction route is generally from $SiO_2$/CaO to calcium silicates (ions) and then to crystalline silicon films.

**Supplementary Table 2 |** The possible reactions occurred during the electrochemical deposition-doping processes for crystalline silicon films in molten calcium chloride[1–7].*

| **I: Electrochemical deposition process** | | |
|---|---|---|
| Compounding reactions | Dissolution reactions | Electrodeposition reactions |
| $x\mathrm{SiO_2} + y\mathrm{CaO} \rightarrow \mathrm{Ca}_y\mathrm{Si}_x\mathrm{O}_{(2x+y)}$ | $\mathrm{Ca}_y\mathrm{Si}_x\mathrm{O}_{(2x+y)} \rightarrow y\mathrm{Ca}^{2+} + \mathrm{Si}_x\mathrm{O}_{(2x+y)}{}^{2y-}$ | $\mathrm{Si}_x\mathrm{O}_{(2x+y)}{}^{2y-} + 4x\mathrm{e}^- \rightarrow x\mathrm{Si} + (2x+y)\mathrm{O}^{2-}$ |
| $SiO_2 + CaO \rightarrow CaSiO_3$ | $CaSiO_3 \rightarrow Ca^{2+} + SiO_3{}^{2-}$ | $SiO_3{}^{2-} + 4e^- \rightarrow Si + 3O^{2-}$ |
| $SiO_2 + 2CaO \rightarrow Ca_2SiO_4$ | $Ca_2SiO_4 \rightarrow 2Ca^{2+} + SiO_4{}^{4-}$ | $SiO_4{}^{4-} + 4e^- \rightarrow Si + 4O^{2-}$ |
| $2SiO_2 + 3CaO \rightarrow Ca_3Si_2O_7$ | $Ca_3Si_2O_7 \rightarrow 3Ca^{2+} + Si_2O_7{}^{6-}$ | $Si_2O_7{}^{6-} + 8e^- \rightarrow 2Si + 7O^{2-}$ |
| $CaSiO_3 + CaO \rightarrow Ca_2SiO_4$ | $CaO \rightarrow Ca^{2+} + O^{2-}$ | |
| $CaSiO_3 + 2CaO + SiO_2 \rightarrow Ca_3Si_2O_7$ | $SiO_2 + O^{2-} \rightarrow SiO_3{}^{2-}$ | |
| $Ca_2SiO_4 + CaO + SiO_2 \rightarrow Ca_3Si_2O_7$ | $SiO_2 + 2O^{2-} \rightarrow SiO_4{}^{4-}$ | |
| $Ca_2SiO_4 + CaSiO_3 \rightarrow Ca_3Si_2O_7$ | $SiO_3{}^{2-} + O^{2-} \rightarrow SiO_4{}^{4-}$ | |
| **II: Electrochemical doping process** | | |
| Compounding reactions | Dissolution reactions | Electrodeposition reactions |
| — | $Ca_3(PO_4)_2 \rightarrow 3Ca^{2+} + 2PO_4{}^{3-}$ | $PO_4{}^{3-} + 5e^- \rightarrow P + 4O^{2-}$ |
| $Sb_2O_3 + x\mathrm{CaO} \rightarrow \mathrm{Ca}_x\mathrm{Sb_2O}_{(3+x)}$ | $\mathrm{Ca}_x\mathrm{Sb_2O}_{(3+x)} \rightarrow x\mathrm{Ca}^{2+} + \mathrm{Sb_2O}_{(3+x)}{}^{2x-}$ | $\mathrm{Sb_2O}_{(3+x)}{}^{2x-} + 6e^- \rightarrow 2Sb + (3+x)O^{2-}$ |
| $Al_2O_3 + x\mathrm{CaO} \rightarrow \mathrm{Ca}_x\mathrm{Al_2O}_{(3+x)}$ | $\mathrm{Ca}_x\mathrm{Al_2O}_{(3+x)} \rightarrow x\mathrm{Ca}^{2+} + \mathrm{Al_2O}_{(3+x)}{}^{2x-}$ | $\mathrm{Al_2O}_{(3+x)}{}^{2x-} + 6e^- \rightarrow 2Al + (3+x)O^{2-}$ |
| $B_2O_3 + CaO \rightarrow CaB_2O_4$ | $CaB_2O_4 \rightarrow Ca^{2+} + B_2O_4{}^{2-}$ | $B_2O_4{}^{2-} + 6e^- \rightarrow 2B + 4O^{2-}$ |

***Note:** We note that the reactions summarized in Supplementary Table 2 are the typical possible reactions, which can only be considered as general guidelines for understanding the electrodeposition process, more detailed reaction mechanisms need further investigation, which are currently being studied. Generally, calcium silicates include $CaSiO_3$, $Ca_2SiO_4$, $Ca_3Si_2O_7$, $Ca_3SiO_5$, *etc*[2]. Calcium aluminates include $CaAl_2O_4$, $CaAl_4O_7$, $Ca_{12}O_{14}O_{33}$, *etc*[7]. Calcium-antimony compounds include $Ca_2Sb_2O_5$, $CaSb_2O_4$, *etc*[5]. Calcium borates mainly include $CaB_2O_4$, *etc*[8].

**Supplementary Table 3 |** Comparison of molten salt electrodeposition process and traditional Siemens process for silicon photovoltaic.

| **Process** | **Steps** | **Temperature (◦C)** | **Materials** |
|---|---|---|---|
| Molten salt electrodeposition technology | One-step: Electrodeposition | 850 | $SiO_2$, CaO, $CaCl_2$ |
| Traditional technology (Siemens process) | Step I: Carbon reduction | About 1700 | $SiO_2$, carbon |
| | Step II: Hydrochlorination | 300 | HCl |
| | Step III: Siemens CVD | 1100 | $H_2$ |
| | Step IV: Crystal growth | 1500 | — |
| | Step V: Wire sawing | 20 | Diamond wire / Waste about 40% of Si |

**Supplementary Note 1 |** The solubility of calcium oxide in molten calcium chloride at 850 ºC is approximately 20 mol. %[1,2]. The solubility of silicon dioxide in molten calcium chloride depends on the concentration of calcium oxide, the dissolution process generally through the reactions, *i.e.*, $SiO_2 + CaO \rightarrow CaSiO_3$; $CaSiO_3 \rightarrow Ca^{2+} + SiO_3^{2-}$; and $SiO_2 + O^{2-} \rightarrow SiO_3^{2-}$, *etc*. It was reported that the solubility of calcium silicate ($CaSiO_3$) in molten calcium chloride electrolyte at 850 ºC is about 1.56 wt. % (within 9 h)[1]. In this work, the solubility of silicon dioxide in molten calcium chloride-calcium oxide electrolyte ($CaCl_2$- about 2 wt. % CaO) was determined to be around 1.5 wt. % (*i.e.*, $CaSiO_3$ about 3.0 wt. %) within approximately 48 hours. The detailed reactions between silicon dioxide and calcium oxide/oxygen ions are listed in Supplementary Table 2.

**Supplementary Note 2 |** The cost analysis in this work is based on a bottom-up model[9-11], by breaking down the total module price into three components, including solar grade silicon (SoG-Si) wafer preparation (polysilicon ingot and wafer slicing), cell fabrication and module assembling. We assume the electrodeposited silicon films are compatible with current technology for cell fabrication and module assembling. So the cost breakdown data for these two steps are obtained directly from publicly available resources. For SoG-Si wafer preparation, the production process is modeled based on aluminum electrolysis, due to the similar reaction mechanism and manufacturing processes. A total cost of ownership model includes the costs associated with materials, labor wages, utilities, facilities, maintenance and depreciation.

The plant locations are chosen all within United States. Labor fees, utility costs, building costs, maintenance costs are all obtained from online resources, assuming an average value for United States.

The module itself is assumed to be 1.96 $m^2$, with 72 cells in each module, with cell thickness of 100 μm.

Comparison (temperature, materials, and steps) of molten salt electrodeposition process and traditional Siemens process for silicon photovoltaic is listed in Supplementary Table 3.

More detailed data that support this cost analysis are available from the corresponding author upon reasonable request.

## Supplementary References


1. Xiao, W. *et al.* Verification and implications of the dissolution-electrodeposition process during the electro-reduction of solid silica in molten $CaCl_2$. *RSC Adv.* **2**, 7588–7593 (2012).

2. Yasuda, K., Nohira, T., Hagiwara, R. & Ogata, Y. H. Diagrammatic representation of direct electrolytic reduction of $SiO_2$ in molten $CaCl_2$. *J. Electrochem. Soc.* **154**, E95–E101 (2007).

3. Zou, X. *et al.* Electrochemical formation of a *p-n* junction on thin film silicon deposited in molten salt. *J. Am. Chem. Soc.* **139**, 16060–16063 (2017).

4. Yang, X. *et al.* Toward cost-effective manufacturing of Si solar cells: Electrodeposition of high quality Si films in a $CaCl_2$-based molten salt. *Angew. Chem. Int. Ed.* **56**, 15078–15082 (2017).

5. Zyryanov, V. V. Mechanochemical synthesis and thermal behavior of metastable mixed oxides in the $CaO$-$Sb_2O_3$-$Bi_2O_3$ system. *Inorg. Mater*. **39**, 1163–1171 (2003).

6. Tacker, R. C. & Stormer Jr, J. C. Thermodynamics of mixing of liquids in the system $Ca_3(PO_4)_2$-$CaCl_2$-$CaF_2$-$Ca(OH)_2$. *Geochim. Cosmochim. Acta*, **57**, 4663–4676 (1993).

7. Yang, S. *et al.* Dissolution-precipitation mechanism of combustion synthesis of calcium aluminate. *Ceram. Int.* **43**, 15918–15926 (2017).

8. Chang, C. -R. & Jean, J. -H. Crystallization kinetics and mechanism of low-dielectric, low-temperature, cofirable $CaO$-$B_2O_3$-$SiO_2$ glass-ceramics. *J. Am. Ceram. Soc*. **82**, 1725–1732 (1999).

9. Song, Z. *et al.* A technoeconomic analysis of perovskite solar module manufacturing with low-cost materials and techniques. *Energy Environ. Sci.* **10**, 1297–1305 (2017).

10 Powell, D. M. *et al*. The capital intensity of photovoltaics manufacturing: barrier to scale and opportunity for innovation. *Energy Environ. Sci.* **8**, 3395–3408 (2015).

11. Sofia, S. E. *et al.* Economic viability of thin-film tandem solar modules in the United States. *Nat. Energy* **3**, 387–394 (2018).
